# Effect of Conditioned Medium from IGF1-Induced Human Wharton’s Jelly Mesenchymal Stem Cells (IGF1-hWJMSCs-CM) on Osteoarthritis

**Published:** 2020

**Authors:** Hanna Sari Widya Kusuma, Wahyu Widowati, Rimonta Febby Gunanegara, Berry Juliandi, Nyoman Ehrich Lister, Seila Arumwardana, Dewani Tediana Yusepany, Dwi Surya Artie, Enden Dea Nataya, Kamila Yashfa Gunawan, Ika Adhani Sholihah, Ermi Girsang, Chrismis Novalinda Ginting, Indra Bachtiar, Harry Murti

**Affiliations:** 1. Biomolecular and Biomedical Research Center, Aretha Medika, Utama, Bandung, West Java, Indonesia; 2. Faculty of Medicine, Maranatha Christian University, Bandung, West Java, Indonesia; 3. Department of Biology, Faculty of Mathematics and Natural Sciences, Bogor Agricultural University, IPB Darmaga Campus, Bogor, West Java, Indonesia; 4. Universitas Prima Indonesia, Medan North Sumatera, Indonesia; 5. Stem Cell and Cancer Institute, Jakarta, Indonesia

**Keywords:** Chondrocyte, IGF1, Osteoarthritis, Proinflammatory, Wharton’s jelly

## Abstract

**Background:**

Osteoarthritis (OA) is a chronic disease that attacks joints and bones which can be caused by trauma or other joint diseases. Stem cell and Conditioned Medium (CM) of stem cells are developed for OA therapy, which is minimally invasive. It can decrease inflammation and be a replacement for knee surgery. This study aimed to utilize human Wharton’s Jelly-Mesenchymal Stem Cells (hWJMSCs) as an alternative OA therapy.

**Methods:**

CM from hWJMSCs induced by IGF1 was collected. The OA cells model (IL1β-CHON002) culture was treated as follows: 1) with hWJMSCs-CM 15% (v/v); 2) with hWJMSCs-CM 30% (v/v); 3) with IGF1-hWJMSCs (IGF1-hWJMSCs-CM) 15% (v/v); 4) with IGF1-hWJMSCs-CM 30% (v/v). Parameters including inflammatory cytokines (IL10 and TNFα), extracellular matrix degradation (MMP3 expression), and chondrogenic marker (*COL2* expression) were determined.

**Results:**

The most significant increase in *COL2* chondrogenic markers was found in IL1β-CHON002 treatment using 15% CM of hWJMSCs induced with IGF1. CM of hWJMSCs can reduce inflammatory cytokines (TNFα and IL10) and matrix degradation mediator MMP3. Better result was gained from IGF1-induced hWJMSCs-CM.

**Conclusion:**

CM of IGF1-hWJMSCs reduce inflammation while repairing injured joint in the human chondrocyte OA model.

## Introduction

The prevalence of Osteoarthritis (OA) and Rheumatoid Arthritis (RA) are increasing in linear to the growth of elderly population ^1^. Osteoarthritis is a chronic disease that attacks joints and bones which is caused by trauma as well as other joint diseases. Commonly, synovial inflammation can cause joint homeostasis disorders related to OA ^2^.

Interleukin-1β (IL1β) is a cytokine that can trigger OA through a variety of mechanisms, such as triggering an imbalance in cartilage repair process, triggering the formation of ROS including Nitric Oxide (NO), inflammatory mediators such as Prostaglandin E2 (PGE2) through increased expression of inducible Nitric Oxide Synthase (iNOS) and Cyclooxygenase-2 (COX2) ^3^. The formation of free radicals and the lack of an antioxidant defense system can trigger oxidative stress causing damage to joints in OA and RA ^4^. Clinical measures for OA therapy are usually based on the main symptoms and the main focus is to reduce pain from inflammation using NonSteroidal Anti-Inflammatory Drugs (NSAIDs) or total replacement of joints ^5^. OA therapy is not intended to regenerate articular cartilage. Continuous use of NSAIDs has side effects that can cause kidney, digestive and cardiovascular disorders ^6,7^.

Chondrocytes which are part of the cartilage are most widely used for OA therapy ^8^; however, treatment using autologous chondrocytes implantation has various disadvantages such as involving two-step surgeries which causes damage and degradation of the cartilage. Mesenchymal Stem Cells (MSCs) have the ability to differentiate into chondrocytes so they are suitable candidates for cartilage regeneration therapy. MSCs have a variety of abilities, one of which is modulating the microenvironment through anti-inflammatory and immunosuppressive functions. Diverse bioactive soluble factors excreted by MSCs can protect cartilage from damage and induce regeneration of remaining progenitor cells ^9^. The ability of homing possessed by MSCs causes MSCs to converge on the cartilage defect and they can proliferate to regenerate articular cartilage, reduce the concentration of synovial fluid from prostaglandins ^10^, and decrease the progressive nature of OA ^11^.

MSCs are first isolated from cartilage [Bone Marrow Mesenchymal Stem Cells (BMMSCs)] and after that they can also be isolated from adipose tissue, placenta, umbilical cord, umbilical cord blood, dental pulp, amnion ^12^ and Wharton’s jelly ^13^. Various therapies for patients with OA are chondroprotective and they reduce inflammation and delay damage to the cartilage ^14^. When compared with BMMSCs, Adipose tissue-MSCs (ADMSCs) have lower chondrogenesis ability. Induction using Transforming Growth Factor-β2 (TGFβ2) and Insulin like Growth Factor-1 (IGF1) in ADMSCs can produce chondrocyte markers comparable to BMMSCs, which include collagen-1A (COL1-A), COL2A1, and SRY-related HMG-box (SOX9) ^15^. Plasmid-based overexpression from IGF1 in rabbit chondrocytes encapsulated using alginate and given *in vivo* shows the ability to repair cartilage and accelerate subchondral bone formation in osteochondral disorders ^16^.

OA treatment uses stem cells, and especially human Wharton’s Jelly Mesenchymal Stem Cells (hWJMSCs) have the potential to be applied in the treatment of OA because of their high regeneration power and are easily obtained because they come from the umbilical cord. The previous study by Sanchooli *et al* shows that conditioned medium from ADMSCs (ADMSCs-CM) has a high potential for bone healing. The effectiveness of MSCs-CM therapy is due to the presence of growth factors and cytokines which can inhibit apoptosis and increase cell proliferation and even stimulate mobilization and placement of stem cells to the site of injury ^17^.

Nevertheless, stem cells transplantation has some obstacles such as differentiation and low cell endurance. These problems can be overcome by using CM obtained from stem cell culture. Analysis shows that CM of hWJMSCs contains various important proteins such as cytokines, growth factors, and angiogenic factors ^18,19^. This study was conducted to evaluate the potential of CM of IGF1-induced hWJMSCs (IGF1-hWJMSCs-CM) for OA therapy.

## Materials and Methods

### Cultivation of hWJMSCs and CM collection

hWJMSCs were collected from the Stem Cell and Cancer Institute (Jakarta, Indonesia). The cells had been characterized by the cell multipotent differentiation and surface phenotype ^13,20^. Informed consent was obtained from the Institutional Ethics Committee at the Stem Cell and Cancer Institute, Jakarta, Indonesia. hWJMSCs at a density of 1×10^6^/well were cultured in Minimum Essential Medium-α (MEM-α) (Gibco, 12561056**)** supplemented with Fetal Bovine Serum (20%) (FBS) (Gibco, 10270106) and 1% antibiotic and antimycotic (Gibco, 1772653). The cells were incubated in a humidified atmosphere with 5% CO_2_ at 37°*C* for 24 *hr*. The medium was discarded and washed with Phosphate Buffered Saline (PBS) (Gibco, 1740576). hWJMSCs at density of 3×10^5^ cells/well were maintained in a complete medium. The cells were treated with IGF-1 (Biolegend, 590904) at concentrations of 0 and 150 *ng/ml*, and incubated at 5% CO_2_, 37°*C* for 7 days, to obtain IGF1-induced hWJMSCs cells (IGF1-hWJMSCs) for measuring *COL2* gene expression. After inducing IGF1, hWJMSCs were harvested. The medium was collected and centrifuged at 3000 *g* for 4 *min* at room temperature, and the supernatant was filtered by a 0.22-*mm* filter (TPP, 99722) and used as CM of hWJMSCs (hWJMSCs-CM) and stored at −80°*C* ^19–21^.

### OA model treated with CM of IGF1-hWJMSCs

Human chondrocyte CHON002 cell line (ATCC RL-2847) (5×10^5^ cells) were obtained from Biomolecular and Biomedical Research Center, Aretha Medika Utama, Bandung, Indonesia. The cells were seeded into T-25 flasks and incubated for 48 *hr*. The medium (hWJMSCs-CM) was replaced and the cells treated with recombinant IL1β (Biolegend, 579404) with concentrations of 0 (no treatment) and 10 *ng/ml* for 5 days in preparation for the OA model ^20–22^.

The experiment were conducted with 6 different groups as follow: 1) CHON002 without IL1β induction and without additions of CM (control); 2) IL1β-CHON002 without additions of hWJMSCs-CM; 3) IL1β-CHON002 treated with hWJMSCs-CM 15% (v/v); 4) IL1β-CHON002 treated with hWJMSCs-CM 30% (v/v); 5) IL1β-CHON002 treated with CM of IGF1-hWJMSCs (IGF1-hWJMSCs-CM) 15% (v/v); 6) IL1β-CHON002 treated with IGF1-hWJMSCs-CM 30% (v/v). The medium were replaced every 2 days. The experiment were carried for 1 and 2 weeks ^21,23^.

### Analysis of COL2 gene expression

RNA was extracted using Aurum RNA kit (Bio-Rad, 7326820) based on the manufacturer’s instructions. The concentration and purity of RNA of each sample was determined at 260/280 *nm* (Table 1). Primer sequences can be seen in table 2. The synthesis of cDNA from the RNA was carried out using iScript cDNA synthesis kit (Bio-Rad, 1708890) at 25°*C* for 5 *min*, 42°*C* for 30 *min*, and 85°*C* for 5 *min* for the final step. The end-product was stored at −20°*C*. Quantitative gene expression was conducted using Thermo Scientific PikoReal Real-time PCR System (Thermo Fisher). PCR included pre-incubation cycle at 95°*C* for 5 *min*, 40 cycles of denaturation at 95°*C* for 1 *min*, annealing at 53°*C* for 40 *s*, and extension at 72°*C* for 1 *min*. The reaction mix that was used to perform qPCR was from Evagreen master mix (Bio-Rad, 1725200). Table 2 shows the primers used in this research ^21,23^.

**Table 1.** Concentration and purity of RNA

**Treatment**	**RNA concentration (*ng/ml*)**		**RNA purity (λ260/λ280 *nm*)**	

	**Week 1**	**Week 2**	**Week 1**	**Week 2**
**Normal cell (CHON002)**	38.32	147.00	2.18	2.29
**IL1β-CHON002**	39.36	162.38	2.13	2.26
**hWJMSCs-CM 15%+IL1β-CHON002**	36.76	134.22	2.10	2.25
**hWJMSCs-CM 30%+IL1β-CHON002,**	61.64	230.02	2.11	2.26
**IGF1-hWJMSCs-CM 15%+IL1β-CHON002**	61.04	233.00	2.13	2.29
**IGF1-hWJMSCs-CM 30%+IL1β-CHON002**	49.48	194.24	2.19	2.27

**Table 2.** Primer sequence of *COL2* and *GAPDH* gene

**Gene symbols**	**Primer sequence (5′ to 3′) upper strand: sense lower strand: antisense**	**Product size (*bp*)**	**Annealing (°*C*)**	**Cycle**	**References**
***COL2***	5′-TTTCCCAGGTCAAGATGGTC-3′ 5′-CTGCAGCACCTGTCTCACCA-3′	377	53	40	NM_001844.4
***GAPDH***	5′-GGGCTGCTTTTAACTCTGGT-3′ 5′-TGGCAGGTTTTTCTAGACGG-3′	702	51	40	NM_001289745.1

### Quantification of IL10, TNFα, and MMP3 level in OA model treated with hWJMSCs-CM

Secretion of IL10, TNFα, MMP3 was assessed using ELISA Kit IL10 (Elabsci, E-EL-H0103), TNFα (Elabsci, E-EL-H0109), and MMP3 (Elabsci, E-ELH1446). The procedure was in accordance with manufacturer’s protocol. Sample absorbances were read at 450 *nm* using microplate reader (Multiskan GO, ThermoScientific). IL10, TNFα, MMP3 concentration were calculated based on a protein standard curve ^19,21,24^.

## Results

### COL2 gene expression level

**Figure 1.**
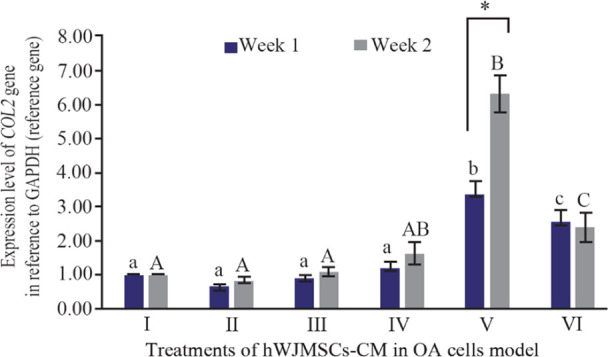
Effect of hWJMSCs-CM, IGF1-hWJMSCs-CM toward *COL2* gene expression on OA cells model. (I) Normal cell (CHON002), (II) IL1β-CHON002, (III) hWJMSCs-CM 15% + IL1β-CHON002 (IV), hWJMSCs-CM 30% + IL1β-CHON002, (V) IGF1-hWJMSCs-CM 15% + IL1β-CHON002, (VI) IGF1-hWJMSCs-CM 30%+IL1β-CHON002. The histograms are presented as mean± standard deviation, the treatment was done triplicate. The data were analyzed with ANOVA and Tukey post hoc test. Different letters (a, b, c) indicate significant differences in 1 week incubation (Blue color) and different letters (A, AB, B, C) indicate significant differences in 2 week incubation (Gray color). The symbol (*) presents significant differences between week 1 and week 2 based on paired t-test (p<0.05).

*COL2* is a monomer protein that forms the main formation of the cartilage matrix and is the main target of tissue that is attacked by OA. The long assembly process of *COL2* in the cytoplasm will eventually be transported out of the cell to form a cartilage matrix and its expression culminates in the ripening of chondrocytes ^25^. *COL2* expression can be seen in figure 1 and has increased from the first week to the second week. The highest expression was found in the IGF1-hWJMSCs-CM 15% treated group.

### Level of TNFα, IL10, and MMP3

**Figure 2.**
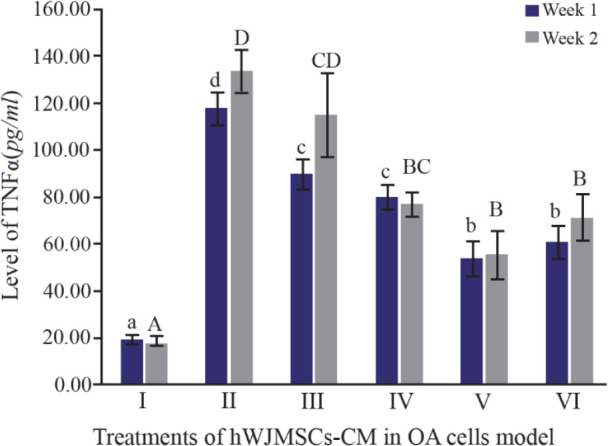
Effect of hWJMSCs-CM, IGF1-hWJMSCs-CM toward TNFα on OA cells model. (I) Normal cell (CHON002), (II) IL1β-CHON002, (III) hWJMSCs-CM 15%+IL1β-CHON002, (IV) hWJMSCs-CM 30%+IL1β-CHON002, (V) IGF1-hWJMSCs-CM 15%+ IL1β-CHON002, (VI) IGF1-hWJMSCs-CM 30%+IL1β-CHON002. The histograms are presented as mean±standard deviation, the treatment was done triplicate. The data were analyzed with ANOVA and Tukey post hoc test. Different letters (a, b, c, d) indicate significant differences in 1 week incubation (Blue color) and different letters (A, B, BC, CD, D) indicate significant differences in 2 week incubation (Gray color).

TNFα, together with IL1β, is considered an inflammatory cytokine which is a key in the pathophysiological process that occurs during OA. TNFα is secreted by the same cells as cells that synthesize IL1β ^26,27^. Figure 2 shows TNFα levels using the ELISA method. It can be seen that IL1β-CHON002 has the highest level of TNFα among others, while addition of CM of IGF1-hWJMSCs 15% shows a significant decrease in TNFα (p<0.05). TNFα also experienced elevated levels in the treatment for 2 weeks.

**Figure 3.**
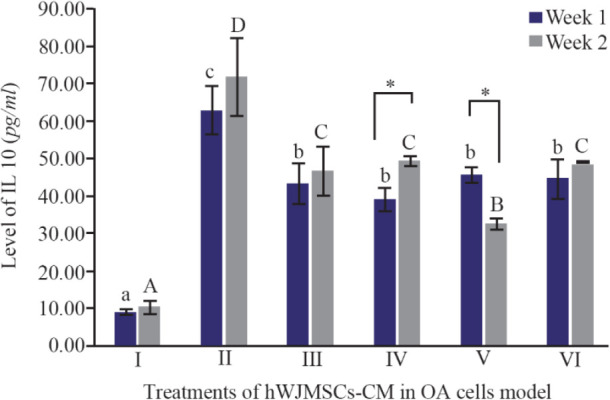
Effect of hWJMSCs-CM, CM of IGF1-hWJMSCs toward IL10 level on OA cells model. (I) Normal cell (CHON002), (II) IL-1β-CHON002, (III) hWJMSCs-CM 15%+IL1β- CHON002 (IV), hWJMSCs-CM 30%+IL1β-CHON002, (V) IGF1-hWJMSCs-CM 15%+IL1β-CHON002, (VI) IGF1-hWJMSCs-CM 30%+IL1β-CHON002. The histograms are presented as mean±standard deviation, the treatment was done triplicate. The data were analyzed with ANOVA and Tukey post hoc test. Different letters (a, b, c) indicate significant differences in 1 week incubation (Blue color) and different letters (A, B, C, D) indicate significant differences in 2 week incubation (Gray color). The symbol (*) presents significant differences between week 1 and week 2 based on paired t-test (p<0.05).

IL10 is a cytokine that acts as an anti-inflammatory agent and it is one of the cytokines that shows a chondroprotective effect on OA ^28^. The IL10 cytokines and IL10R receptors are expressed by chondrocytes ^29^. IL10 works by stimulating antagonist proteins against IL1β, namely IL1Ra, metalloproteinase inhibitors (TIMP1), and also as growth factors. IL10 levels can be seen in figure 3. IL10 as an anti-inflammatory mediator was found with the highest levels in IL1β-CHON002. However, addition of hWJMSCs-CM with or without IGF1 induction shows a decrease in IL10 levels compared to OA cells model.

**Figure 4.**
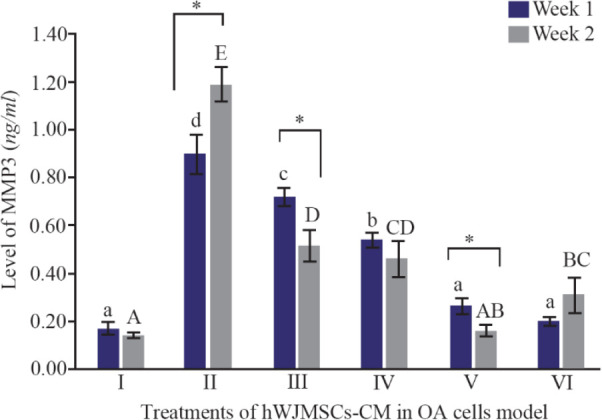
Levels of MMP3 in cells of OA models cultured in hWJMSCs-CM. (I) Normal cell (CHON002), (II) IL1β-CHON002, (III) hWJMSCs-CM 15%+IL1β-CHON002 (IV), hWJMSCs-CM 30%+IL1β-CHON002, (V) IGF1-hWJMSCs-CM 15%+IL1β-CHON002, (VI) IGF1-hWJMSCs-CM 30%+IL1β-CHON002. The histograms are presented as mean ± standard deviation, the treatment was done triplicate. The data were analyzed with ANOVA and Tukey post hoc test. Different letters (a, b, c, d) indicate significant differences in 1 week incubation (Blue color) and different letters (A, AB, BC, C, CD, D, E) indicate significant differences in 2 week incubation (Gray color). The symbol (*) presents significant differences between week 1 and week 2 based on paired t-test (p<0.05).

The MMPs are expressed in joint tissues of patients with OA and RA. The MMP3 is secreted from chondrocyte and synovial cells and MMP3 can reduce various extracellular matrix. Figure 4 shows MMP3 levels using ELISA method. The lower MMP3 level was shown by treatment with the addition of 15%, 30% of IGF1-hWJMSC-CM during 1 and 2 weeks of incubation, while the lowest MMP3 levels was during 2 weeks of incubation of IGF1-hWJMSCs-CM 15%. This result shows that IGF1-hWJMSCs-CM can reduce levels of MMP3 which plays a role in matrix degradation.

## Discussion

MSCs have been used as one of the candidates for tissue engineering which have the ability in repair, replacement, and regeneration of cartilage tissue, because of their high proliferation and differentiation ^29^. One type of them originates from Wharton’s jelly ^13,19^. Previous studies reported that hWJMSCs can differentiate into chondrocytes ^21,24^, skeletal muscle cells, heart muscle cells, osteoblasts, adipocytes, β cells on the islets of Langerhans, and endothelial cells *in vitro* ^30,31^. Therefore, these cells can be used as alternatives in the treatment of chronic degenerative disorders and prevent cartilage degradation in OA patients. In this study, the induction of hWJMSCs using IGF1 increased *COL2* expression. The *COL2* gene which is the cartilage matrix gene has been indicated to be regulated by SOX9 ^32,33^, and is involved in the structure and function of articular cartilage. This is in accordance with previous studies, where the inducing of IGF1 in hWJ-MSCs increased the expression of the *SOX9* gene which means that the expression of the *COL2* gene will also increase ^21^. *SOX9* is present in the presumed cartilage during embryonic development. *COL2* downregulation in OA is likely to contribute to cartilage pathology. IGF1 can stabilize the chondrocyte phenotype in pathological conditions, and also has mitogenic properties in the articular cartilage of adults and strongly stimulate the production of chondrocyte extracellular matrix components ^21^. *COL2* is a chondrogenic gene marker in the joint cartilage, associated with Extracellular Matrix Secretion (ECM). In OA, early changes in articular cartilage are characterized by proteoglycan loss and a decrease in the expression of the *COL2* gene without altering the regularity of the articular tissue structure ^34^. After treatment, as shown in figure 1, there were significant improvements in *COL2* gene expression in OA model, indicating that there might be repairing process in the ECM. The highest expression of *COL2* is by IGF1-hWJMSCs-CM 15%+IL1β-CHON-002 which is significantly different from other treatments. It is known that the expression of *COL2* is closely related to *SOX9*.

The effect of TNFα in many cases coincides with the action of IL1β, and a relationship can be detected that occurs during OA between two cytokines ^28^. This effect is the result of activating the same group of intracellular signaling pathways, which in turn triggers effects that increase inflammation in joint tissues ^35^. In contrast, IL10 is a cytokine that acts as an anti-inflammatory agent and it is one of the cytokines that shows a chondroprotective effect on OA and works by stimulating antagonist proteins against IL1β as pro-inflammatory cytokine ^28^.

In the present study, the highest levels of TNFα were found in OA cells without treatment using CM of hWJMSCs and IGF1-hWJMSCs. This is possible because the production of proinflammatory cytokines is directly proportional to the production of anti-inflammatory cytokines, when high levels of proinflammatory cytokines occur and the body adjusts to provide a regulatory response to levels of anti-inflammatory cytokines. Furthermore, hWJMSCs-CM and IGF1-hWJMSCs-CM in the OA cells model decrease TNFα level which is responsible for OA inflammation; however, administration of them to IL10 did not show an increase in IL10 levels responsible for inhibiting pro-inflammatory cytokines. This is in line with the results of Al-Banna *et al* who state that the induction of inflammatory cytokines is followed by an increase in the level of anti-inflammatory cytokines ^36^.

Cell therapies can directly aid repair by forming new functional tissues, or support tissue repair through paracrine mechanisms, for instance by secreting growth factors, immunomodulatory molecules, and Extracellular Vesicles (EVs). EVs can mediate cell-cell communication and are involved in many processes, including immune signaling, angiogenesis, stress response, senescence, proliferation, and cell differentiation ^36^. *In vitro* passaging of MSCs results in cell enlargement, differentiation, and decrease in proliferation within 10 passages, and causes a strong response to micro-environment stiffness, affecting cell morphology, and function ^37,38^.

Metabolic imbalances between degradation and synthesis of articular cartilage are the main reason for degeneration in OA sufferers. MMP is a protein that is responsible for the degradation of the extracellular matrix and basement membrane components ^39^. MMP is a endopeptidase that is connected with zinc ions and is localized in various connective tissues; it can degrade various components of the ECM ^40^. In the present study, OA model (IL1β-CHON002) has shown high MMP3 levels, and after treatment using hWJMSCs-CM and IGF1-hWJMSCs-CM, showed a decrease in MMP3 levels (Figure 4) and the lowest MMP3 level was shown by treatment with the addition of IGF1-hWJMSCs-CM 30%. This indicates that the administration of hWJMSCs-CM with the addition of IGF1 can reduce MMP3 levels which are the cause of the degradation of the extracellular matrix.

## Conclusion

In conclusion, CM of IGF1-induced hWJMSCs increases *COL2* gene expression compared with CM of IGF1-uniduced hWJMSCs. CM of IGF1-hWJMSCs actively reduce the levels of pro-inflammatory cytokines of TNFα, IL10, MMP3 compared with CM of IGF1-uniduced hWJMSCs. CM of IGF1-hWJMSCs also increases chondrogenesis and can subsequently be an alternative for the treatment of OA. Further studies on animal models must be carried out for validation of IGF1-hWJMSCs effect.
